# Clinical features and prognosis of patients co-infected with HIV and tuberculosis in the ICU

**DOI:** 10.1186/cc12943

**Published:** 2013-11-05

**Authors:** Ana Carla Pecego, Camila Ribeiro da Silva, Rodrigo T Amâncio, Denise M Medeiros, Emerson C Mesquita, André M Japiassú, Fernando A Bozza

**Affiliations:** 1 Instituto de Pesquisa Clínica Evandro Chagas, Fundação Oswaldo Cruz, RJ, Brazil

## Background

Despite the advances in treatment, tuberculosis (TB) remains a global health threat and is the leading co-infection among Brazilian HIV-infected patients. Mortality of TB in the presence of sepsis and shock septic is still high, with respiratory failure being the leading cause of ICU admission. Clinical factors enhancing mortality among HIV-TB patients are yet to be explored.

## Materials and methods

We retrospectively accessed data of HIV/AIDS critically ill patients from January 2007 until May 2012, at a referral infectious diseases hospital. All patients admitted to the ICU with laboratory-confirmed tuberculosis were included in the analysis. Demographic and clinical data were categorized for survivors and nonsurvivors and the results were displayed as frequency (%), median values and interquartile range.

## Results

Fifty patients were included. Hospital mortality was 48%. Age was 31.5 years (26.5 to 43) in the survivors group versus 33.5 years (30 to 45) in nonsurvivors (*P *= 0.25), and SAPS II score was 44.5 (37 to 56) versus 47 (39 to 54) (*P *= 0.58). The most common TB presentation was disseminated disease (56%) followed by pulmonary (44%), with no difference according to survival. The interval of days between ICU admission and TB treatment was not different between groups. Rifampicin (94%), pyrazynamide (94%), isoniazide (92%) and ethambutol (76%) were administered in the majority of patients, while fluoroquinolones and aminoglycosides were administered in 64% and 54% respectively. Nonsurvivors presented with more elapsed time since HIV diagnosis (1 (1 to 7) vs. 26 (5 to 72), *P *= 0.10); lower nadir CD4 cell count (72.5 (27.5 to 133.5) vs. 24 (14.5 to 63), *P *= 0.03); and HAART initiated within 30 days after admission (62% vs. 29%, *P *= 0.03; odds ratio 3.9 (95% CI 1.2 to 12.7)). The main reason for ICU admission was respiratory failure (70%). Nonsurvivors needed mechanical ventilation (88% vs. 48%, *P *< 0.01) and vasopressors (71% vs. 41%, *P *= 0.05) more frequently. Neurological dysfunction was more common in nonsurvivors (79% vs. 41%, *P *= 0.01, odds ratio 5.2 (95% CI 1.5 to 18.2)). After multivariate analysis, neurological dysfunction was associated with hospital mortality, while HAART in the first 30 days of hospitalization was a protective associated factor.

## Conclusions

Disseminated TB was the most common presentation in HIV/AIDS critically ill patients. Nonsurvivors were more prone to multiple organ dysfunction syndrome, and neurological dysfunction was associated with hospital mortality. The administration of HAART within 30 days of hospitalization was associated with survival.

